# Myeloid-specific ferritin light chain deletion does not exacerbate sepsis-associated AKI

**DOI:** 10.1152/ajprenal.00043.2024

**Published:** 2024-05-23

**Authors:** James D. Odum, Juheb Akhter, Vivek Verma, Giacynta Vollmer, Ahmad Davidson, Kelly A. Hyndman, Subhashini Bolisetty

**Affiliations:** ^1^Division of Pediatric Critical Care, Department of Pediatrics, University of Alabama at Birmingham, Birmingham, Alabama, United States; ^2^Division of Nephrology, Department of Medicine, University of Alabama at Birmingham, Birmingham, Alabama, United States

**Keywords:** acute kidney injury, ferritin, inflammatory response, sepsis

## Abstract

Sepsis-associated acute kidney injury (SA-AKI) is a key contributor to the life-threatening sequelae attributed to sepsis. Mechanistically, SA-AKI is a consequence of unabated myeloid cell activation and oxidative stress that induces tubular injury. Iron mediates inflammatory pathways directly and through regulating the expression of myeloid-derived ferritin, an iron storage protein comprising ferritin light (FtL) and ferritin heavy chain (FtH) subunits. Previous work revealed that myeloid FtH deletion leads to a compensatory increase in intracellular and circulating FtL and is associated with amelioration of SA-AKI. We designed this study to test the hypothesis that loss of myeloid FtL and subsequently, circulating FtL will exacerbate the sepsis-induced inflammatory response and worsen SA-AKI. We generated a novel myeloid-specific FtL knockout mouse (FtL^LysM–/–^) and induced sepsis via cecal ligation and puncture or lipopolysaccharide endotoxemia. As expected, serum ferritin levels were significantly lower in the knockout mice, suggesting that myeloid cells dominantly contribute to circulating ferritin. Interestingly, although sepsis induction led to a marked production of pro- and anti-inflammatory cytokines, there was no statistical difference between the genotypes. There was a similar loss of kidney function, as evidenced by a rise in serum creatinine and cystatin C and renal injury identified by expression of kidney injury molecule-1 and neutrophil gelatinase-associated lipocalin. Finally, RNA sequencing revealed upregulation of pathways for cell cycle arrest and autophagy postsepsis, but no significant differences were observed between genotypes, including in key genes associated with ferroptosis, an iron-mediated form of cell death. The loss of FtL did not impact sepsis-mediated activation of NF-κB or HIF-1a signaling, key inflammatory pathways associated with dysregulated host response. Taken together, while FtL overexpression was shown to be protective against sepsis, the loss of FtL did not influence sepsis pathogenesis.

**NEW & NOTEWORTHY** Hyperferritinemia in sepsis is often associated with a proinflammatory phenotype and poor prognosis. We previously showed the myeloid deletion of FtH results in a compensatory increase in FtL and is associated with reduced circulating cytokines and decreased rates of SA-AKI in animal sepsis models. Here, we show that myeloid deletion of FtL does not impact the severity of SA-AKI following CLP or LPS, suggesting that FtH plays the predominant role in propagating myeloid-induced proinflammatory pathways.

## Graphical abstract

**Figure:**
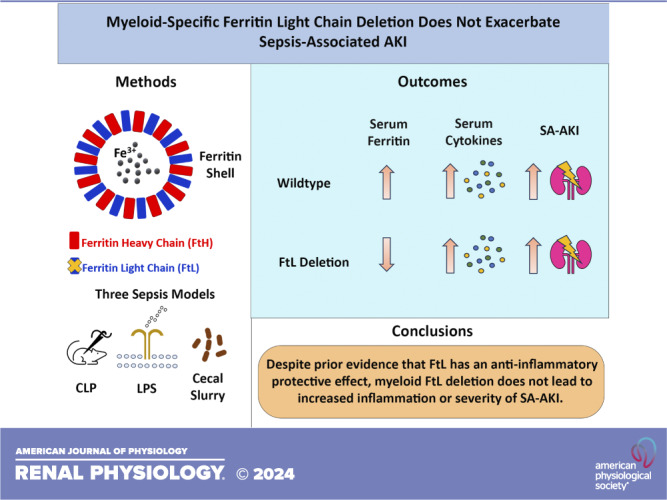

## INTRODUCTION

Iron is a potent mediator of the proinflammatory cascade after a host is challenged with an immune-activating stimulus, such as injury, ischemia, or infection. Circulating iron is primarily stored in a heteropolymeric ferritin shell complex, which consists of iron, ferritin heavy chain (FtH), and ferritin light chain (FtL) (1). FtH is primarily responsible for oxidizing ferrous iron to its ferric form, whereas FtL serves to nucleate ferric iron inside the ferritin shell (2). FtH and FtL are ubiquitously expressed under homeostatic conditions and abundantly stored in tissue such as the liver, spleen, and skeletal muscle (3).

During perturbations of homeostasis, macrophages and other myeloid-derived immune cells modulate the ratio of circulating ferritin-iron complexes to intracellularly sequestered iron to carefully orchestrate a balanced immune response to injury (4). In addition, iron is preferentially sequestered from the blood into tissues and stored in intracellular ferritin complexes to prevent pathogens from using iron to proliferate in the setting of infection (5). However, mechanisms of iron handling can become dysregulated, resulting in proinflammatory states that induce multiorgan failure with high rates of mortality (6–8). The kidneys are particularly vulnerable to injury from disturbances in iron handling, given the high metabolic demands required to achieve adequate filtration and solute clearance (9).

Previous animal studies demonstrate that iron and FtH contribute to the activation of proinflammatory pathways, manifesting as increased levels of interleukins (IL)-1β, IL-6, IL-8, and tumor necrosis factor-α (TNF-α) (7, 10–12). On the contrary, studies have also demonstrated a potent role for ferritin in regulating the inflammatory response (8, 13–15). In previous work, we demonstrated that targeted deletion of myeloid-FtH expression leads to a reduction in levels of systemic inflammation, organ injury, and mortality following sepsis. However, myeloid-FtH deletion also correlated with a compensatory increase in intracellular myeloid FtL expression and elevated circulating ferritin levels (10). The degree to which FtL influences inflammatory-mediated AKI remains unclear. In this study, we used murine models of sepsis to interrogate the role of FtL in the pathophysiology of sepsis-associated acute kidney injury (SA-AKI). Current evidence suggests FtL overexpression, in the setting of sepsis, may attenuate the severity of proinflammatory pathways and protect against SA-AKI (10). Therefore, in a myeloid-specific FtL knockout model, we hypothesize that selective deletion of FtL will exacerbate hyperinflammatory pathways, leading to increased systemic proinflammatory cytokines and more severe patterns of SA-AKI in mice challenged with sepsis.

## MATERIAL AND METHODS

### Experimental Model

Male and female mice aged 10–14 wk were used in this study. We generated a transgenic mouse on the C57BL/6 background in which the endogenous FtL gene (exons 1–4) were flanked by loxP sites (FtL^fl/fl^). To generate transgenic mice with targeted deletion of FtL in the myeloid compartment (FtL^LysM–/–^), we bred the floxed mice with LysM Cre mice (Cat. No. 4781, Jackson Laboratories). All procedures involving mice were performed in accordance with National Institutes of Health guidelines regarding the care and use of live animals and were reviewed and approved by the Institutional Animal Care and Use Committee of UAB.

### Confirmation of Myeloid FtL-Deficient Mice

To confirm myeloid FtL deficiency, DNA was isolated from bone marrow-derived macrophages (BMDMs) and tails of mice and subjected to PCR with the primers mentioned in Table 1.

**Table 1.** Primers used for genotyping mice

Primer Name	Gene Target	Primer Sequence
F4	FtL	5′-GCCTTGCTACCCCTTCCACTA-3′
R4	FtL	5′-GTCAGTCTTACGCAGAACAGTTGG-3′
F5	FtL	5′-GGTGGAACACCGGACAATACTGC-3′
R5	FtL	5′-CAACCCTCCCGTTATCAATC-3′

### Cecal Ligation and Puncture-Induced Sepsis

Mice were anesthetized using inhaled isoflurane (1.5–2% isoflurane induction, 1–1.5% maintenance). Their lower abdomen was incised (0.5 cm) to expose the intraperitoneal cavity under aseptic conditions. The cecum was located, and 5-0 monofilament nylon suture was used to ligate the cecum 0.7 cm from the tip. A single through-and-through pass was made in the cecum using a 21-gauge needle, and bowel contents were expressed into the peritoneal cavity. The ligated cecum was returned to the intraperitoneal cavity, and the peritoneum and skin were sutured using 4-0 nylon monofilament nonabsorbable suture. Mice were injected with 1 mL of normal sterile saline via subcutaneous injection for resuscitation. Mice were closely monitored with unrestricted access to water and food and euthanized 24 h after the surgery. Blood was collected via cardiac puncture, and plasma was isolated by a 1,500 *g* 10-min centrifugation, and kidneys, liver, and spleen were harvested for subsequent analysis.

### Lipopolysaccharide-Induced Sepsis

Mice were anesthetized using isoflurane (1.5–2% isoflurane induction, 1–1.5% maintenance) and administered two different doses of lipopolysaccharide (LPS) (Cat. No. L2630, Sigma-Aldrich) by intraperitoneal (IP) injection. We administered LPS at 8 mg/kg body weight to model severe sepsis or 2.5 mg/kg body weight to model mild sepsis as a means to validate our findings across the spectrum of sepsis severity. All mice were euthanized 24 h after the injection. Blood and tissues were collected as outlined earlier.

### Cecal Slurry-Induced Sepsis

Cecal slurry (CS) stock was generated from 14-wk-old male C57BL/6 mice (Cat. No. 0664, Jackson Laboratories, Bar Harbor, ME). Donor mice underwent terminal anesthesia via inhaled isoflurane (2.5–3.5%) followed by cervical dislocation at the end of the procedure. After the mice were fully anesthetized, a midline laparotomy was performed under aseptic conditions. The cecum was isolated and excised; stool contents were manually expressed and collected into a single stock preparation. Cecal stool was suspended in a ratio of 100-mg stool per 1 mL of sterile phosphate-buffered saline (PBS) + 5% glycerol solution to generate CS (16). CS was filtered first through a sterile 860-µm mesh followed by filtering through a sterile 190-µm mesh (Bellco Glass, Inc.) and then stored at −80°C until the day of sepsis induction. We determine a CS dose corresponding to a 50% mortality rate at 48 h (LD_50_) for each CS stock generated (17).

The female littermates of FtL^fl/fl^ and FtL^LysM–/–^ male mice aged 10–14 wk were used for sepsis induction via CS. To induce sepsis, we administered a CS dose corresponding to the LD_50_ via IP injection; for this study, the CS LD_50_ was 0.5 mg/g body weight prepared in sterile PBS to achieve a total volume of 200 µL for IP injection. Mice were euthanized at 24 h post-CS and blood and tissues were collected as stated earlier.

### Cell Culture of Bone Marrow-Derived Macrophages

Bone marrow-derived macrophages (BMDMs) were isolated and cultured as previously described (18). Briefly, murine femurs were flushed and disintegrated into a single-cell suspension and passed through 40-μm cell strainer (BD Falcon). Red blood cells were lysed using ACK lysis buffer (ThermoFisher). The remaining cells were incubated in Dulbecco’s modified Eagle’s medium (Corning Cell Gro) with 10% fetal bovine serum (Atlanta Biologicals), 30 ng/mL MCSF (MiltenyiBiotec), and 1% penicillin/streptomycin (Life Technologies). BMDMs were stimulated with 100 ng/mL of LPS for varying durations (0, 0.5, 1, 3, 6, and 24 h).

### Peritoneal Colony Forming Unit Measurement

Peritoneal lavage was performed at the onset of harvest by administering 1 mL of sterile 1× PBS + 5% glycerol via IP injection followed by abdominal massage to adequately mixed peritoneal fluid, and then aspiration of peritoneal fluid was performed. Peritoneal lavage (50 μL) was cultured using serial dilutions onto 3.7% wt/vol brain-heart infusion broth with 0.15% wt/vol agar plates and incubated at 37°C for 24 h. Colonies were counted, and data were represented as Log_10_ colony forming unit (CFU) per milliliter.

### Complete Blood Cell Count Profiling

Whole blood was obtained from cardiac puncture using a heparinized syringe and processed fresh at room temperature. Blood cell composition was analyzed using a Veterinary Hematology Analyzer (Element HT5, Heska) for the following parameters: red blood cell (RBC) count, white blood cell (WBC) count, lymphocyte (LYM), monocyte (MON), neutrophil (NEU), eosinophils (EOS), basophils (BAS), hemoglobin (Hb), hematocrit (HCT), mean corpuscular volume (MCV), mean corpuscular hemoglobin (MCH), mean corpuscular hemoglobin concentration (MCHC), RBC distribution width (RDW), platelet count (PLT), and mean platelet volume (MPV).

### Serum or Plasma Analysis

For serum or plasma analysis, blood was collected by cardiac puncture as described earlier. Serum or plasma was obtained from the blood and centrifuged at 5,000 rpm for 10 min. Ferritin (Cat. No. KT-396, Kamiya Biomed) and Cystatin C (Cat. No. MSCTC0, R&D Systems) were measured in the serum according to the manufacturer’s instructions. Creatinine was measured by LC-MS/MS.

### Serum Cytokine Measurement

Serum cytokine measurement was done using a mouse V-PLEX Pro-inflammatory Panel I Kit (Cat. No. K15048D, Meso Scale Discovery) according to the manufacturer’s instructions. A MESO Sector S600 plate reader (Meso Scale Discovery) was used for data acquisition. Cytokine levels are reported as picograms per milliliter.

### Western Blot

Harvested tissues and cells were lysed in Lysis Buffer (10 mM Tris·HCl, 5 mM EDTA, 150 mM NaCl, 10% Nonidet P-40, and 10% Triton-X) with endogenous protease (Sigma-Aldrich) and phosphatase (Cat. No. A32955, Sigma-Aldrich) inhibitors. Lysates were subjected to centrifugation at 15,000 *g* for 10 min at 4°C. Clear supernatant was collected and quantified by bicinchoninic acid protein assay (Cat. No. 23227, ThermoFisher) according to the manufacturer’s instructions. Total protein (cells: 15–20 µg; tissue: 75 µg) was resolved on 12% Tris-glycine sodium dodecyl sulfate polyacrylamide gel electrophoresis or readymade 4–12%, BIS-TRIS polyacrylamide gel (Cat. No. NW04127BOX, ThermoFisher), and then transferred on to a polyvinylidene fluoride membrane (Cat. No. 1PVH00005, Millipore). Membranes were blocked following the manufacturer’s instructions (5% non-fat dry milk in Tris-buffered saline with 0.1% Tween (TBST) or 5% BSA in TBST) for 1 h at room temperature. After blocking, membranes were probed with desired primary antibodies such as goat anti-kidney injury molecule-1 (KIM-1) (Cat. No. AF1817, R&D Systems, 1:500), goat anti-neutrophil gelatinase-associated lipocalin (NGAL) (Cat. No. AF1857, R&D Systems, 1:2,000), mouse anti-FtL (Cat. No. sc-74513, Santa Cruz, 1:1,000), mouse anti-p-P65 (Cat. No. sc-136548, Santa Cruz, 1:1,000), rabbit anti-total P65 (Cat. No. 4764S, Cell Signaling, 1:1,000), rabbit anti-HIF-1 (Cat. No. 10006421, Cayman, 1:1,000), rabbit anti-HO-1 antibody (Cat. No. SPA-894-F, Enzo LifeSciences, 1:1,000) followed by incubation with peroxidase-conjugated anti-mouse (Cat. No. R1005, Kindle Biosciences, 1:1,000), anti-rabbit (Cat. No. R1006, Kindle Biosciences, 1:1,000), and anti-goat (Cat. No. R1007, Kindle Biosciences, 1:1,000) antibodies, respectively. Horseradish peroxidase activity was detected using chemiluminescence (Cat. No. R1002, KwikQuant) detection system. Membranes were stripped and reprobed with a mouse anti-GAPDH antibody (Cat. No. MAB374, Millipore, 1:10,000) to ensure equal loading and transfer. Densitometry was performed using ImageJ software, and results were normalized to GAPDH and total P65 in the case of P-p65.

### Prussian Blue Staining

Transverse kidney, liver, and spleen sections from FtL^fl/fl^ and FtL^LysM–/–^ baseline mice were fixed in 10% neutral-buffered formalin for 24 h, then transferred to 70% ethanol before embedding in paraffin and cutting into 5-µm sections. Tissue sections were deparaffinized with xylene, passed through decreasing concentrations of ethanol (100%>95%>70%), and then rehydrated in water. The sections were then immersed in a solution of equal parts hydrochloric acid and potassium ferrocyanide for 20 min, followed by three washes in water before incubating in nuclear fast red for 5 min. After this, the sections were rinsed twice in water and then dehydrated through increasing concentrations of ethanol (70–100%) and xylene, and finally mounted. Images were captured using a Keyence BZ-X800 microscope.

### Immunohistochemistry

Transverse spleen sections from FtL^fl/fl^ (*n* = 6) and FtL^LysM–/–^ (*n* = 8) baseline mice were fixed in 10% neutral-buffered formalin for 24 h, then transferred to 70% ethanol before embedding in paraffin and cutting into 5-µm sections. Tissue sections were deparaffinized with xylene, passed through decreasing concentrations of ethanol (100%, 95%), and then rehydrated in water before endogenous peroxidase inactivation with 3% H_2_O_2_ (Cat. No. ca37722-84-1, Fisher Bioreagent) for 30 min at room temperature. Antigen retrieval was performed by steaming sections in citrate buffer (pH = 6.0) for 35 min. Sections were then blocked in 2.5% normal horse serum (Vector Laboratories) for 20 min at room temperature in a humidified chamber, followed by incubation with primary antibody against FtL (10727-1-AP, Proteintech) in 2.5% normal horse serum (Vector Laboratories) overnight at 4°C in a humidified chamber. The following day, sections were washed in 10 mM PBS before labeling with secondary antibody horse anti-rabbit (Cat No. MP-7401, Vector Laboratories) or horse anti-goat (Vector Laboratories), respectively, for 30 min at room temperature in a humidified chamber. Sections were washed in PBS and developed with the 3,3′-diaminobenzidine substrate kit (Cat. No. SK-4105, Vector Laboratories) for 5 min. Tissue sections were dehydrated through increasing concentrations of ethanol (70–100%) and xylene, and then mounted. Images were captured using a Keyence BZ-X800 microscope.

### Bulk RNA Sequencing

Total RNA was extracted from half of a kidney from each mouse using the Direct-Zol RNA miniprep kit (Cat. No. R2052, Zymo Research, Irvine, CA). RNA was lyophilized in RNA stabilization tubes (Azenta Life Sciences, Burlington, MA) and shipped to Azenta for RNA sequencing. cDNA libraries were generated, including poly A selection (Illumina, San Diego, CA), and they were sequenced on the Illumina HiSeq3000/4000 in a 2 × 150 base pair configuration, targeting 30 million reads per sample.

Our bulk RNA sequencing analysis used RStudio and the R environment (v. 4.2.1).

The FASTQ file quality was checked using FASTQC in the package Trim_Galore!, and adapter sequences were trimmed. File quality was checked again with FASTQC, and the files were then mapped to the mouse genome (mm10) with the program STAR. The BAM files generated were then used to determine raw counts with featureCounts in the package subread. Finally, these count matrixes were used in the R package DESeq2 to determine DEGs. The entire dataset was used to generate the normalized counts, and the following pairwise comparisons were then made: WT baseline versus WT LPS treatment, KO baseline versus KO LPS treatment, and WT LPS versus KO LPS.

We performed a targeted analysis of inflammatory and iron/ferritin-mediated pathways in our bulk RNA sequencing data using Gene Ontology (GO), the Kyoto Encyclopedia of Genes and Genomes (KEGG) analysis, and Reactome through DAVID Bioinformatics (19–21). GOplot was used to generate visual representations of these key pathways (22).

### Statistics

Data are represented as means ± SE. An unpaired two-tailed student’s *t* test was performed for comparisons between two groups. For comparison between more than two groups, analysis of variance (ANOVA) followed by Tukey’s post hoc analysis was used. *P* < 0.05 was considered significant. Survival was determined by Kaplan–Meier curve. GraphPad Prism 7 was used for all statistical analysis.

## RESULTS

### Characterization of Myeloid FtL Deletion

**Figure 1.**
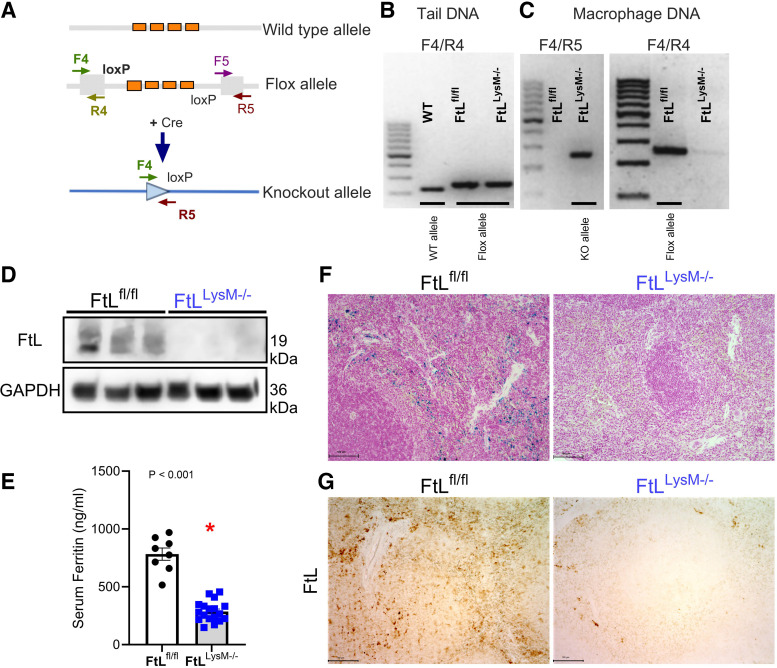
Characterization of selection myeloid ferritin light (FtL) manipulation in FtL^fl/fl^ (wild-type floxed) and FtL^LysM−/−^ (knockout) mice. *A*: schematic of primer positions used in confirmation of FtL deletion. *B*: tail DNA from wild-type (C57BL/6), FtL^fl/fl^, and FtL^LysM−/−^ mice was subjected to PCR with a F4/R4 primer pair that demonstrated the presence of a flox allele in FtL^fl/fl^ and FtL^LysM−/−^ mice, thus confirming the selective myeloid FtL deletion. PCR with Cre primers confirmed the presence of Cre in FtL^LysM−/−^ mice. *C*: DNA isolated from cultured bone marrow-derived macrophages (BMDMs) from FtL^fl/fl^ and FtL^LysM−/−^ mice was used for amplification with the F4/R5 primer pair confirming deletion of the FtL gene in FtL^LysM−/−^ mice but not in FtL^fl/fl^ mice. We used the F4/R4 primer pair to confirm the loxP site in wild-type FtL^fl/fl^ mice; BMDM DNA from FtL^LysM−/−^ mice did not show amplification due to deletion of FtL. *D*: Western blot from BMDMs confirming retained FtL expression in FtL^fl/fl^ mice and lack of FtL expression in FtL^LysM−/−^ mice. *E*: serum ferritin was lower in FtL^LysM−/−^ mice compared with wild-type FtL^fl/fl^ mice. *F*: Prussian blue staining revealing myeloid FtL deletion (FtL^LysM−/−^) results in less splenic iron retention compared with wild-type mice. *G*: FtL^fl/fl^ mice had more robust splenic FtL abundance than their FtL^LysM−/−^ counterparts. All data are expressed as means ± SE. *P < 0.05 vs. FtL^fl/fl^(*n* = 8 FtL^fl/fl^, 18 FtL^LysM-/-^).

We validated the deletion of ferritin light chain specifically from the myeloid compartment of (FtL^LysM–/–^) mice (Fig. 1). A schematic representation of the loxP site and primer positions used to confirm FtL deletion are shown in Fig. 1*A.* Littermate FtL floxed mice (FtL^fl/fl^) and myeloid-specific FtL-deficient (FtL^LysM–/–^) mice were used for this study. PCR of tail genomic DNA from wildtype (C57BL/6), FtL^fl/fl^, and FtL^LysM–/–^ mice revealed the presence of the floxed allele only in the transgenic mice (Fig. 1*B*). In addition, the Cre transgene was detected only in tail DNA from FtL^LysM–/–^ mice. PCR analysis of genomic DNA from bone marrow-derived macrophages (BMDMs) revealed deletion of the FtL gene only in macrophages from FtL^LysM–/–^ mice (Fig. 1*C*). Next, we confirmed that FtL protein was reduced in BMDMs of FtL^LysM–/–^ mice compared with wildtype (Fig. 1*D*), and this correlated with reduced serum ferritin levels in FtL^LysM–/–^ mice (Fig. 1*E*). We also found that FtL deletion was associated with markedly reduced splenic iron content in the knockout mice (Fig. 1*F*). Further examination of livers and kidneys revealed that there was no compensatory iron deposition in these organs of the knockout mice (Supplemental Fig. S2). In addition, immunohistochemistry was performed on spleens from unstressed FtL^fl/fl^ and FtL^LysM–/–^ mice and confirmed the deletion of myeloid FtL (Fig. 1*G*). FtL^LysM–/–^ mice were born at the expected Mendelian ratio and did not manifest any apparent abnormalities. Given the role of myeloid FtL in iron handling, we performed complete blood cell analysis but found no significant difference between unstressed mice FtL^fl/fl^ and FtL^LysM–/–^ mice (Table 2). Taken together, this validates the deletion of myeloid FtL in the FtL^LysM–/–^ mice.

**Table 2.** Effects of deletion of myeloid FtL on the morphological blood parameter changes during sepsis-induced acute kidney injury in mice

	Baseline		CLP	
Parameter	FtL^fl/fl^	FtL^LysM−/−^	FtL^fl/fl^	FtL^LysM−/−^
RBC (10^6^/μL)	8.456 ± 0.11	8.929 ± 0.10	8.928 ± 0.26	8.451 ± 0.28
WBC (10^3^/μL)	2.635 ± 0.50	4.432 ± 0.48	1.146 ± 0.14*	1.159 ± 0.28*
LYM (10^3^/μL) MON (10^3^/μL)	1.401 ± 0.26 0.065 ± 0.01	2.672 ± 0.40 0.113 ± 0.02	0.625 ± 0.09 0.073 ± 0.01	0.421 ± 0.07 0.072 ± 0.02
NEU (10^3^/μL)	0.926 ± 0.24	1.467 ± 0.32	0.293 ± 0.05	0.257 ± 0.08*
EOS (10^3^/μL)	0.22 ± 0.10	0.144 ± 0.03	0.127 ± 0.04	0.319 ± 0.13
BAS (10^3^/μL)	0.023 ± 0.007	0.036 ± 0.005	0.028 ± 0.007	0.09 ± 0.052
Hb (g/dL)	13.25 ± 0.20	13.95 ± 0.21	14.01 ± 0.35	13.45 ± 0.37
HCT, %	39.72 ± 0.60	42.08 ± 0.51	41.97 ± 1.08	40.16 ± 1.22
MCV (fL)	46.95 ± 0.20	47.11 ± 0.15	47.07 ± 0.42	47.59 ± 0.44
MCH (pg)	15.67 ± 0.10	15.61 ± 0.11	15.73 ± 0.11	15.96 ± 0.20
MCHC (g/dL)	33.38 ± 0.11	33.14 ± 0.15	33.4 ± 0.19	33.57 ± 0.47
RDW (%)	13.78 ± 0.31	13.89 ± 0.27	12.77 ± 0.26*	13.43 ± 0.42
PLT (10^3^/μL)	512.4 ± 140.38	499.4 ± 110.9	307.4 ± 52.04	452.7 ± 75.35
MPV (fL)	5.55 ± 0.20	5.38 ± 0.19	5.8 ± 0.16	5.76 ± 0.15

BAS, basophils; EOS, eosinophils; HCT, hematocrit; Hb, hemoglobin; LYM, lymphocyte; MCH, mean corpuscular hemoglobin; MCHC, mean corpuscular hemoglobin concentration; MCV, mean corpuscular volume; MON, monocyte; MPV, mean platelet volume; NEU, neutrophil; PLT, platelet count; RBC, red blood cell; WBC, white blood cell. Significant differences between groups are designated as follows: *P < 0.05 vs. baseline.

### Deletion of Myeloid FtL Does Not Impact Mortality or Systemic Inflammation After CLP

**Figure 2.**
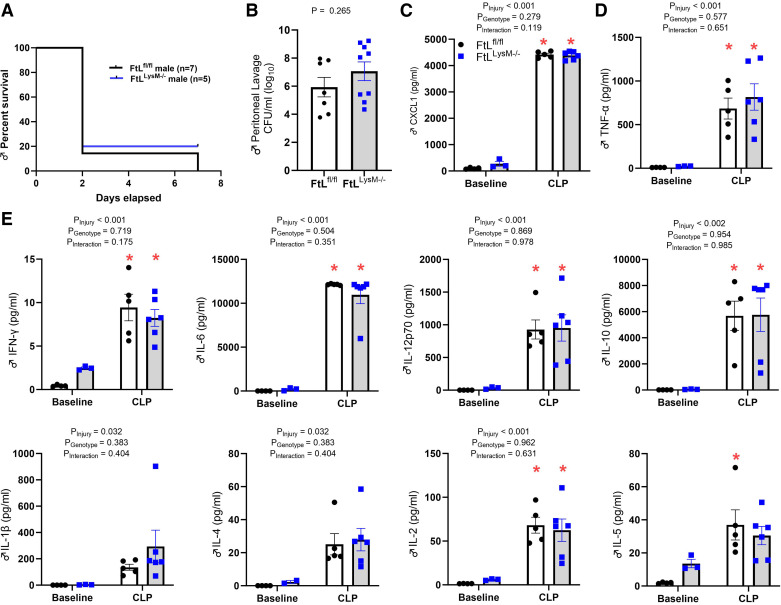
*A*: deletion of myeloid ferritin light (FTL) did not impact mortality or sepsis-induced kidney injury. Shown is a Kaplan–Meier graph indicating percent survival of FtL^fl/fl^ and FtL^LysM−/−^ mice following cecal ligation and puncture (CLP). *B*: peritoneal bacterial burden at 24 h following CLP as quantified by log_10_ colony forming units (CFU) in peritoneal lavage of FtL^fl/fl^ and FtL^LysM−/−^ mice. *C–E*: plasma levels of TNF-α, CXCL1, IL-12, IL-6, IL-5, IL-4, IL-2, IL-1β, IFN-γ, and IL-10 were measured 24 h after CLP. All data are expressed as means ± SE. *P < 0.05 vs. baseline.

Given our previously published data demonstrating the deletion of myeloid FtH dampens systemic proinflammatory cytokines, decreases acute kidney injury during sepsis, and is associated with a concurrent compensatory increase in circulating FtL (10), we hypothesized that inflammation would be exacerbated after deletion of myeloid FtL. Following CLP, FtL^fl/fl^ and myeloid FtL-deficient FtL^LysM–/–^ mice experienced nearly 80% mortality by 48 h, with no significant difference between genotypes (Fig. 2*A*). There was no difference in peritoneal bacterial clearance by genotype, as measured by quantification of bacterial burden in peritoneal lavage following CLP (Fig. 2*B*). We performed a serum cytokine multiplex analysis at 24 h following CLP and observed a significant increase in both proinflammatory cytokines such as TNF-α, interferon-γ (IFN-γ), IL-6, IL-12, chemokine CX-C ligand-1 (CXCL1), IL-10, IL-2, IL-5, and the anti-inflammatory cytokine IL-10 in FtL^fl/fl^ and FtL^LysM–/–^ mice after CLP compared with baseline. However, no significant difference was found in between genotypes (Fig. 2, *C*–*E*).

### Myeloid FtL Deletion Does Not Impact Systemic Blood Cell Counts or Cytokine Production at 24 h Following CLP

We performed a complete blood cell analysis at 24 h following CLP and compared hematological parameters to baseline values (Table 2). There was a general reduction in white blood cell counts, absolute lymphocyte counts, and absolute neutrophil counts in both genotypes following CLP. However, myeloid-FtL deletion did not result in any significant differences in hematological parameters between FtL^fl/fl^ and FtL^LysM–/–^ mice at 24 h following CLP.

### Myeloid FtL Deletion Does Not Impact Markers of Sepsis-Associated Acute Kidney Injury at Early Time Points Following CLP

**Figure 3.**
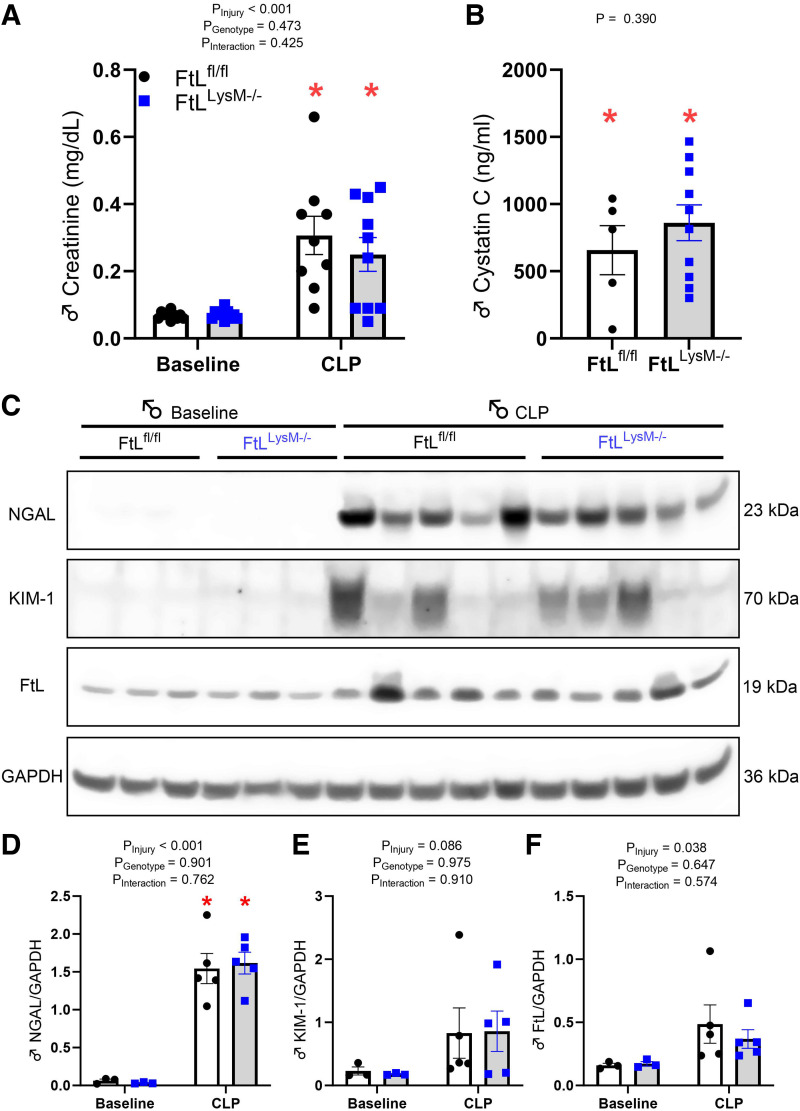
*A*: myeloid ferritin light (FtL) deletion does not impact sepsis-associated acute kidney injury after cecal ligation and puncture (CLP). Plasma creatinine levels were elevated at 24 h post-CLP, but no differences were observed between genotypes. *B*: plasma cystatin C was elevated in FtL^LysM−/−^ mice at 24 h following CLP relative to baseline, but no difference was observed between genotypes. *C–F*: FtL^fl/fl^ and FtL^LysM−/−^ mouse kidneys were collected 24 h after CLP (*C*), and lysates were analyzed for expression of NGAL (*D*), KIM-1 (*E*), and FtL (*F*) by densitometry, normalized to glyceraldehyde-3-phosphate dehydrogenase (GAPDH) and expressed in arbitrary units (AU). GAPDH was used as a loading control. All data are expressed as means ± SE. *P < 0.05 vs. baseline.

At 24 h post-CLP, both genotypes demonstrated a significant rise in serum creatinine and cystatin C levels compared with baseline (Fig. 3, *A* and *B*), but no differences were observed between genotypes. Renal expression of NGAL and KIM-1, which are well-established markers for acute kidney injury (23), was significantly higher in FtL^fl/fl^ and FtL^LysM–/–^ mice 24 h after CLP compared with baseline. However, no significant difference was observed between the genotypes (Fig. 3, *C*–*E*). Despite myeloid FtL deletion, we observed increased bulk kidney FtL expression in both FtL^fl/fl^ and FtL^LysM–/–^ mice 24 h post-sepsis compared with quiescence, which could be attributed to kidney parenchymal cell FtL expression. However, we did not find a significant difference in FtL expression between genotypes (Fig. 3*F*).

### Serum Ferritin Is Elevated Following LPS Administration

**Figure 4.**
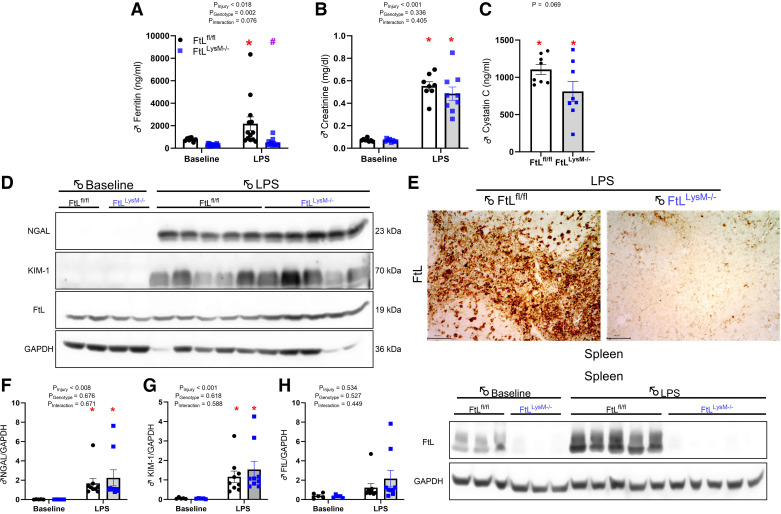
*A*: myeloid ferritin light (FtL) deletion did not impact sepsis-associated acute kidney injury after lipopolysaccharide (LPS). Serum ferritin was reduced in FtL^LysM−/−^ mice at 24 h post-LPS compared with FtL^fl/fl^ mice. Serum creatinine (*B*) and cystatin C (*C*) levels were elevated following LPS administration. *D*: kidney injury markers were detected 24 h after LPS administration and compared with untreated mice (baseline) in kidney lysates. *E*: splenic tissue from both FtL^fl/fl ^and FtL^LysM-/- ^mice 24 h post-LPS treatment was probed for FtL via immunohistochemistry, demonstrating a depletion of FtL from the myeloid compartment under LPS challenge in FtL^LysM-/-^ mice. KIM-1, NGAL, and FtL, were probed on the same membranes after stripping. Expression of NGAL (*F*), KIM-1 (*G*), and FtL (*H*) in the kidneys was analyzed by densitometry, normalized to glyceraldehyde-3-phosphate dehydrogenase (GAPDH) and expressed in arbitrary units (AU). All data are expressed as means ± SE. *P < 0.05 vs. baseline. *#P* < 0.05 vs. FtL^fl/fl^.

In corroboration with clinical and preclinical studies (1, 10, 24, 25), we found that sepsis caused a significant rise in systemic ferritin levels, an effect that was lost in the FtL^LysM–/–^ mice (Fig. 4*A*). These findings underscore the role of myeloid cells in contributing to circulating ferritin levels during sepsis and further indicate that circulating ferritin is predominantly made up of FtL. Myeloid FtL deficiency does not impact LPS-induced acute kidney injury.

There was a significant increase in serum creatinine and cystatin C in both FtL^fl/fl^ and FtL^LysM–/–^ mice following LPS administration at a dose of 8 mg/kg (Fig. 4, *B* and *C*). However, no differences in serum creatinine were observed between genotypes at 24 h after either LPS dose. KIM-1 and NGAL expression were also elevated in the kidneys of mice after LPS administration, but no differences were seen between genotypes (Fig. 4, *D*, *F*, and *G*). Finally, kidney FtL expression was increased in both genotypes following LPS (Fig. 4*H*) suggesting that non-myeloid FtL expression may mediate the protective effect in the kidney. We also examined the expression of FtL in the spleen after LPS induction to confirm that FtL was deleted from the myeloid compartment. We also found an increase in FtL protein levels in FtL^fl/fl^ mice that were administered LPS (Fig. 4*I*). This was further confirmed by immunohistochemical staining of spleens. We also validated FtL deletion from the myeloid compartment of FtL^LysM–/–^ mice (Fig. 4*E*). We further recapitulated these findings using a mild form of sepsis (LPS administered at 2.5 mg/kg), suggesting that FtL deletion did not affect sepsis pathogenesis (Supplemental Fig. S3).

### Selective Myeloid FtL Deletion Alters Systemic Cytokines Following LPS

**Figure 5.**
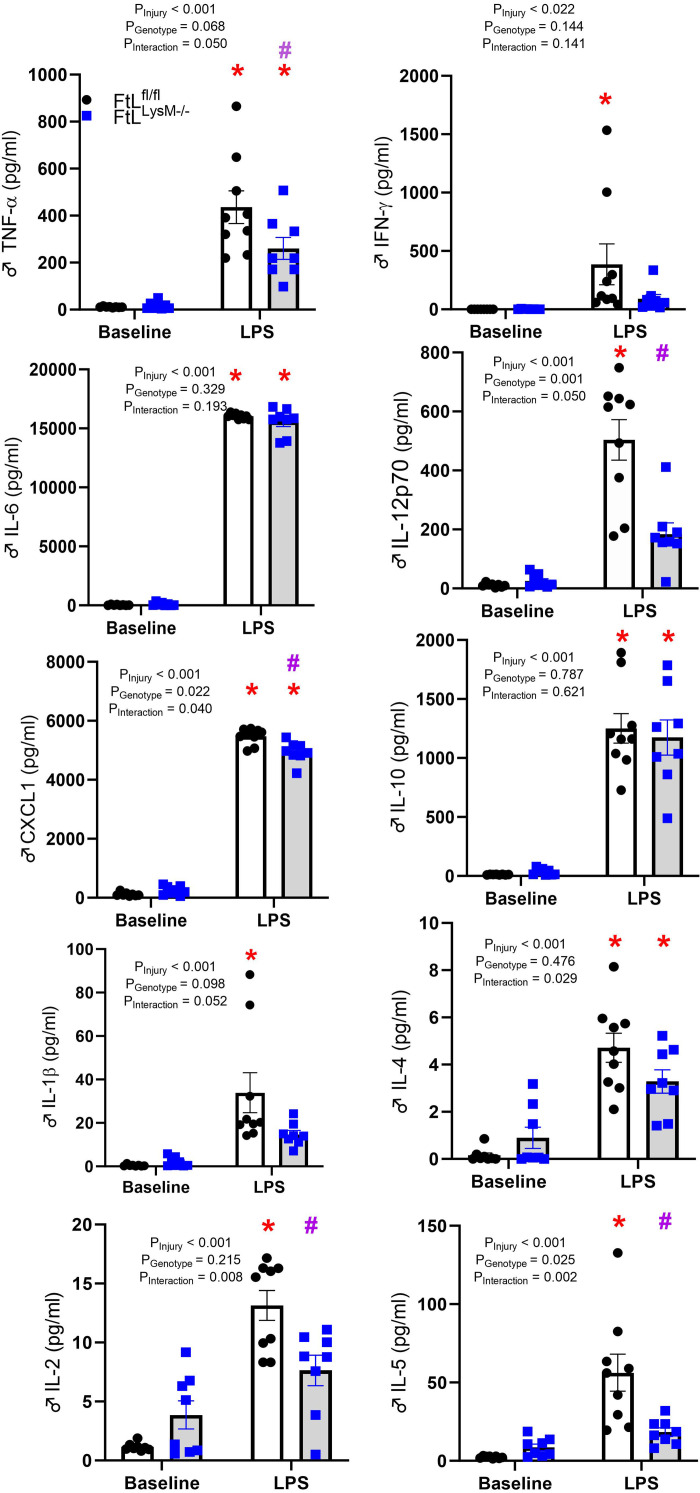
Myeloid ferritin light (FtL) deficiency differentially impacts cytokines following lipopolysaccharide (LPS) administration. Serum levels of IL-6, IFN-γ, IL-4, CXCL1, IL-1β, TNF-α, IL 10, IL-5 IL-12, and IL-2 were measured in untreated (baseline) mice and 24 h following LPS administration. Data (in pg/mL) are expressed as means ± SE. *P < 0.05 vs. baseline. *#P* < 0.05 vs. FtL^fl/fl^.

We analyzed serum cytokines at baseline and 24 h after 8 mg/kg LPS administration in both FtL^fl/fl^ and FtL^LysM–/–^ mice. LPS administration induced systemic cytokine production in both genotypes compared with baseline, with FtL^fl/fl^ demonstrating a significant increase in CXCL1, TNF-α, IL-5, IL-12, and IL-2 compared with FtL^LysM–/–^ mice (Fig. 5). LPS dosed at 2.5 mg/kg generated increases in serum levels of pro- and anti-inflammatory cytokines, but IL-1β was the only cytokine with significantly lower induction in FtL^LysM–/–^ mice compared with wildtype mice (Supplemental Fig. S4).

### Cecal Slurry Did Not Induce SA-AKI Despite Generation of Systemic Inflammation

We administered CS via IP injection to female FtL^fl/fl^ and FtL^LysM–/–^ littermates as a third sepsis model. Similar to CLP, CS administration induced neutropenia, lymphopenia, and thrombocytopenia at 24 h relative to genotype-matched female control mice (Supplemental Table S1). Plasma cytokines were also elevated following CS, particularly IL-6 and CXCL1, but no significant genotypic differences were observed (Supplemental Fig. S1*C*). Despite the expected induction of systemic inflammation, there was no significant elevation in plasma creatinine or cystatin C levels in either genotype (Supplemental Fig. S1, *A* and *B*).

### Bulk Kidney RNA Sequencing Indicates LPS Promotes Similar Cell Cycle Arrest and Autophagy Pathways Despite Myeloid FtL Deletion

**Figure 6.**
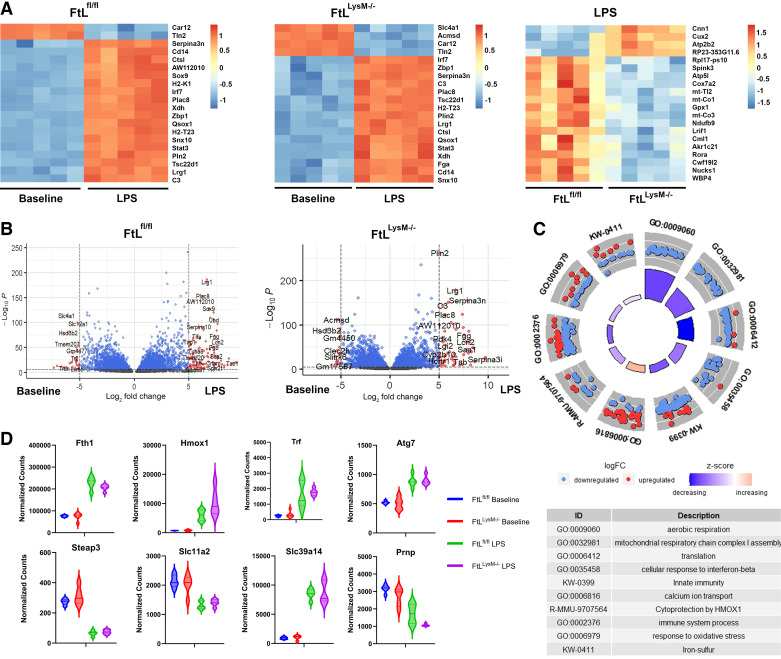
*A*: bulk kidney sequencing data confirmed acute kidney injury state, but myeloid ferritin light (FtL deletion) did not interfere with renal ferroptosis. Shown are the top 20 differentially expressed genes (DEGs) in FtL^fl/fl^ mice that received lipopolysaccharide (LPS) (compared with baseline), FtL^LysM−/−^ mice that received LPS (compared with baseline), and FtL^LysM−/−^ vs. FtL^fl/fl^ at 24 h following LPS administration. *B*: volcano plots representing top DEGs in FtL^fl/fl^ and FtL^LysM-/-^ mice following LPS compared with their respective genotypic baselines. *C*: differences in key pathways in FtL^LysM−/−^ (“upregulated”) versus FtL^fl/fl^ (“downregulated”) at 24 h following LPS administration. Pathways were manually selected based on statistical significance and consolidation of similar terms or pathways. *D*: expression levels of key genes involved in ferroptosis across FtL^fl/fl^ baseline, FtL^LysM−/−^ baseline, FtL^fl/fl^ at 24 h following LPS, and FtL^LysM−/−^ at 24 h following LPS.

We performed bulk RNA sequencing on kidneys from FtL^fl/fl^ and FtL^LysM–/–^ mice at baseline and at 24 h following injection with LPS. Comparing FtL^fl/fl^ and FtL^LysM–/–^ mice injected with LPS to their respective baselines, similar genes and pathways associated with cytokine signaling, cellular response to hypoxia, and apoptosis were upregulated at 24 h. Among the top 20 differentially expressed genes, there was considerable overlap in key gene expression with upregulation of *Serpina3n, C3, Cd14, Ctsl, Irf7, Plac8, Xdh, Zbp1, Qsox1, H2-T23, Snx10, Tsc22d1, and Lrg1,* and downregulation of *Cart12* and *Tln2* in both genotypes after LPS challenge (Fig. 6, *A* and *B*). This gene expression pattern aligns with proinflammatory pathways associated with type 1 interferon and TGF-β production that promotes cell cycle arrest and autophagy.

When directly comparing FtL^LysM–/–^ versus FtL^fl/fl^ mice 24 h following injection of LPS, FtL^LysM–/–^ knockout mice demonstrated downregulation of pathways associated with aerobic respiration, protein translation, and cellular response to interferon-β compared with wildtype FtL^fl/fl^ mice (Fig. 6*C*). The genes with the highest differential expression in the kidneys of FtL^LysM–/–^ mice after LPS were *Cnn1, Atp2b2, and Cux2; Cnn1 and Atp2b2* serve roles in smooth muscle contraction and calcium regulation, respectively. Gene expression in the kidneys of FtL^fl/fl^ mice after LPS was notable for expression of *Lrif1, Spink3, and Gpx1*—all of which have various functions related to cell survival.

We also sought to characterize ferroptosis-related gene expression differences between FtL^LysM–/–^ mice at baseline and following LPS challenge to determine the potential impact of myeloid-specific FtL deletion on iron-regulated cell death. Although there were no significant differences between FtL^fl/fl^ and FtL^LysM–/–^, we observed significant upregulation of transferrin (*Trf*), ZIP14 (*Slc39a14*), *Hmox1*, FtH (*Fth1*), and *Atg7* in mice challenged with LPS (Fig. 6*D*). Likewise, a significant downregulation of *Steap3,* DMT1 (*Scl11a2*), and *Prnp* was observed. These transcriptional changes potentially correspond to decreased cellular trafficking of Fe^2+^ into the cytoplasm as well as downregulation of pathways that reduce Fe^3+^ to Fe^2+^. This might reflect negative feedback in upstream mediators of ferroptosis secondary to the degree of inflammation and cellular injury inflicted by LPS.

### Myeloid FtL Deficiency Does Not Impact NF-κB and HIF-1 Signaling

**Figure 7.**
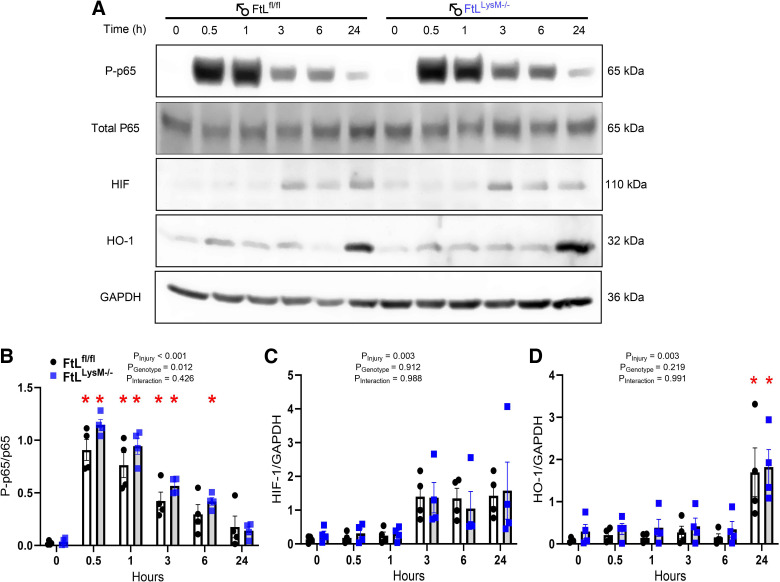
*A*: myeloid ferritin light (FtL) deletion did not impact NF-κB or HIF-1 signaling in bone marrow-derived macrophages (BMDMs). BMDMs of wild-type (FtL^fl/fl^) and FtL-deficient (FtL^LysM−/−^) mice were collected at 0, 0.5, 1, 3, 6, and 24 h after lipopolysaccharide (LPS) stimulation and probed for expression of phosphorylated-p65 (P-p65), total p65, hypoxia inducible factor-1 (HIF-1), and heme oxygenase-1 (HO1). Densitometry analysis was done by using glyceraldehyde-3-phosphate dehydrogenase (GAPDH) or total p65 for normalization. *B–D*: densitometry graphs for P-p65 (*B*), HIF-1 (*C*), and HO-1 (*D*). A representative Western blot from one out of four mice is shown here. Data are presented as means ± SE. *P < 0.05 vs. unstimulated (0 h).

To assess the impact of myeloid FtL deletion on myeloid inflammatory and HIF-1 signaling, we quantified the phosphorylation of the P65 subunit of NF-κB and HIF-1 protein in cultured BMDMs at different time points following LPS stimulation. Phosphorylation of P65 (P-p65) was induced after LPS stimulation at 0.5 h time point and diminished by 24 h (Fig. 7, *A* and *B*). However, the degree of phosphorylated P65 was not significantly different between FtL^fl/fl^ and FtL^LysM–/–^ mice. HIF-1 protein expression was detected as early as 3 h post-LPS stimulation that persisted until 24 h (Fig. 7, *A* and *C*). However, there was no significant difference in HIF1 abundance between FtL^fl/fl^ and FtL^LysM–/–^ at any time point. Finally, we assessed levels of hemeoxygenase-1 (HO-1) following LPS stimulation and found that expression peaked at 24 h post-LPS stimulation with no differences observed between genotypes (Fig. 7, *A* and *D*).

## DISCUSSION

Ferritin plays a critical role in regulating the inflammatory response through the biological activity of FtH and FtL in murine models of sepsis. Although previous work has suggested that FtL is capable of dampening the inflammatory cascade associated with FtH activity (10), the results of this study suggest that myeloid-specific knockout of FtL does not alter systemic proinflammatory cytokine production or the degree of acute kidney injury in mice challenged with CLP or LPS.

There is growing evidence to suggest that hyperferritinemia is not simply a harbinger of the underlying proinflammatory sepsis cascade, but instead represents the direct contribution of aberrant myeloid activation, cytokine generation, and systemic tissue injury to the development of multiorgan dysfunction in sepsis (26). Beyond labeling ferritin as an acute phase reactant, animal models have demonstrated direct mechanistic ties between ferritin and end-organ injury in sepsis (24, 27, 28). In particular, FtH has been linked to the development of multiorgan failure in mouse models of sepsis. Mice lacking myeloid FtH have improved survival, reduced hepatic injury, and stabilization of hemodynamic measurements compared with wild-type controls following CLP (10). In addition, FtH has been shown to induce macrophage polarization independent of iron levels in vitro (29). However, measured circulating ferritin is predominantly comprised of FtL, despite having a less well-delineated biological role in regulating inflammation or promoting ferroptosis (30). Characterization of hereditary hyperferritinemia-cataract syndrome reveals a predominant increase in FtL with normal FtH levels and no significant signals of proinflammatory pathway activation at quiescence despite the presence of hyperferritinemia (31). Likewise, repression of FtL increases the propensity for ferroptosis in a lung adenocarcinoma model, indicating a potential counterregulatory role for FtL in balancing the proinflammatory activity of FtH (32). Despite these findings, direct evidence for FtL as an anti-inflammatory mediator in sepsis remains elusive.

As part of this study, we generated a novel transgenic mouse strain that leverages the myeloid-specific LysM promoter to delete FtL production in macrophages, monocytes, and other myeloid cells (33). Deletion of FtL did not result in any significant changes in FtH myeloid expression, but it did reduce the levels of circulating serum ferritin in unstressed animals compared with circulating ferritin levels in wildtype FtL^fl/fl^ mice. Notably, there was no difference in kidney FtL expression between genotypes at baseline, likely due to the ongoing expression of FtL in kidney epithelial cells despite myeloid FtL deletion (34). Our findings further confirm that the reticuloendoethelial system is primarily responsible for circulating ferritin production (30).

Despite hypothesizing that FtL deletion in myeloid cells would result in exacerbation of proinflammatory pathways resulting in a higher degree of kidney injury, this does not appear to be the case. We suspect that FtL production by kidney epithelial cells can partially compensate for the lack of systemic FtL in circulation during times of stress. We also used a severe model of CLP that resulted in 80% mortality by 48 h post-injury, which may exceed what is salvageable by any anti-inflammatory properties inherent to FtL. However, our findings with two different doses of LPS with varying severity of inflammation and kidney injury further confirm that myeloid FtL deletion does not affect sepsis pathogenesis. Despite evidence of compensatory increase in FtL in previous studies (10), there is a distinct possibility that the absence of FtH was sufficient to reduce inflammation, and any observed increase in FtL had a neutral effect on the inflammatory cascade.

This study was impacted by a few notable limitations. First, sepsis is a challenging injury profile to model in rodents. There is often considerable variability within the same sepsis model due to the complex interplay of host-pathogen interactions, as well as biological factors attributable to age and sex differences. We attempted to interrogate our myeloid FtL knockout model through broad exposure to multiple sepsis models, but consistently demonstrated that myeloid FtL deletion did not influence kidney injury. It should be noted, however, that while myeloid FtL deletion significantly lowered circulating ferritin levels, it did not lead to complete ablation of serum ferritin, suggesting that non-myeloid cells may contribute to circulating ferritin in mice. Thus, it is also possible that the knockout mice did not exhibit an exaggerated hyperinflammatory response following sepsis induction due to the presence of low levels of serum ferritin levels in these mice. Second, we were unable to directly assess the degree by which FtL deletion in LysM-expressing myeloid compartment impacted the FtL expression in kidney resident macrophages. Presumably, there may be differences in FtL expression between kidney resident macrophages and infiltrating macrophages due to the embryonic seeding of resident macrophages that generally self-renew within the kidney (35). In addition to kidney epithelial cell production of FtL, kidney resident macrophages may also be able to express FtL and compensate for LysM-specific myeloid FtL deletion. The expression of FtL in non-myeloid cell populations limits our ability to unequivocally conclude that FtL does not have a protective anti-inflammatory role in SA-AKI. Nevertheless, we can conclude that the absence of FtL from myeloid cells does not significantly alter systemic inflammation or renal tubular injury in SA-AKI. Finally, this study only examined the effects of myeloid FtL deletion on kidney injury at early time points. It is biologically plausible that the kidney maintains a degree of redundancy in times of stress independent of FtL activity, but that chronic disease states or multiple acute injuries might differentially overcome the renal reserve observed in this study.

### Perspectives and Significance

Myeloid-specific FtL deletion does not alter the systemic production of proinflammatory cytokines or early markers of acute kidney injury in mice challenged with sepsis. Taken together with previous work, this amplifies FtH’s role as the predominant ferritin subunit responsible for inducing myeloid polarization and activation of hyperinflammatory pathways. Future studies should consider FtL manipulation in non-myeloid cell populations, such as kidney epithelial cells as well as further delineate the impact of myeloid FtL deletion on chronic or multiple-hit acute injury models to add clarity on its role as an anti-inflammatory counterbalance to FtH.

## DATA AVAILABILITY

The data for this study are available upon reasonable request. The bulk kidney RNA sequencing data have been uploaded to the Gene Expression Omnibus (GSE255281).

## SUPPLEMENTAL DATA

10.6084/m9.figshare.25696272 Supplemental Figs. S–S4 and Supplemental Table S1: https://doi.org/10.6084/m9.figshare.25696272.

## GRANTS

This work was supported by the National Institutes of Health (Grant RO1DK122986 to S.B.) and the American Heart Association (Grant 24POST1200957 to J.A.).

## DISCLOSURES

No conflicts of interest, financial or otherwise, are declared by the authors.

## AUTHOR CONTRIBUTIONS

J.D.O., J.A., and S.B. conceived and designed research; J.D.O., J.A., V.V., G.V., and A.D. performed experiments; J.D.O., J.A., V.V., and K.A.H. analyzed data; J.D.O., J.A., and S.B. interpreted results of experiments; J.A. and G.V. prepared figures; J.D.O., J.A., G.V., and S.B. drafted manuscript; J.D.O., K.A.H., and S.B. edited and revised manuscript; J.D.O., J.A., V.V., G.V., A.D., K.A.H., and S.B. approved final version of manuscript.
